# Unusual acquired gastric outlet obstruction during infancy: a case report

**DOI:** 10.1186/1757-1626-1-237

**Published:** 2008-10-15

**Authors:** PK Srivastava, AN Gangopadhyay, VD Upadhyaya, SP Sharma, R Jaiman, V Kumar

**Affiliations:** 1 Department of Pediatric Surgery, Institute of Medical Sciences, Banaras Hindu University, Varanasi, 221005, U.P. India

## Abstract

Acquired gastric outlet obstruction (GOO) during infancy beyond the neonatal period is a very rare condition when other congenital causes like infantile hypertrophic pyloric stenosis, antral diaphragm, pyloric atresia etc are excluded. We report an unusual case of 6 month old male child who presented with recurrent episode of vomiting not relieved by medication. On gastrograffin study there was pre pyloric stricture of unknown etiology and was managed by stricturoplasty. We are reporting this case because of its rarity and with excellent outcome if diagnosed and managed properly. Even on extensive search of English literature we are not able to find a single report of this lesion in infants.

## Background

Gastric outlet obstruction (GOO) in infancy and childhood may result from congenital causes [antral diaphragm, pyloric atresia, and infantile hypertrophic pyloric stenosis(IHPS)], or acquired causes (peptic ulcer, caustic ingestion, tumor, chronicgranulomatous disease, and eosinophilic gastroenteritis) [3,4]. Among them, IHPS is the most common cause with an incidence of up to 1.5–3 per 1,000 live births. When IHPS is excluded, however, the other causes of GOO in children are relatively rarely encountered, and the incidence of the latter causes was only one in 100,000 live births [2]. IHPS can be diagnosed easily and respond well to Ramstedt's Pyloromyotomy. Other causes of gastric outlet obstruction are; pyloric atresia, prepyloric webs, and diaphragm which can be managed by excision of membrane and pyloroplasty. The prepyloric stricture of unknown etiology is very rare in infants, and it should be kept in mind when IHPS is ruled out.

## Case report

A 6 month old male child weighing 4.2 kg presented to our institute with continuous projectile nonbilious vomiting for the last two months. The frequency of vomiting was gradually increased in last one month. The baby was on medical treatment (ante-emetics) but the frequency of vomiting was not controlled. There was no history of caustic ingestion & analgesic intake. The child lost significant weight during this period. On physical examination child was lethargic and severely dehydrated. On examination there was fullness in epigastrium with visible peristalsis. Palpation does not reveal any added finding. On laboratory investigation the child was anemic with hypokalemic hyponatraemic alkalosis. Blood urea and serum creatinine was within normal limit. Child was managed with nasogastric aspiration and intravenous fluid and antibiotics till the serum electrolyte correction.

**Figure 1:**
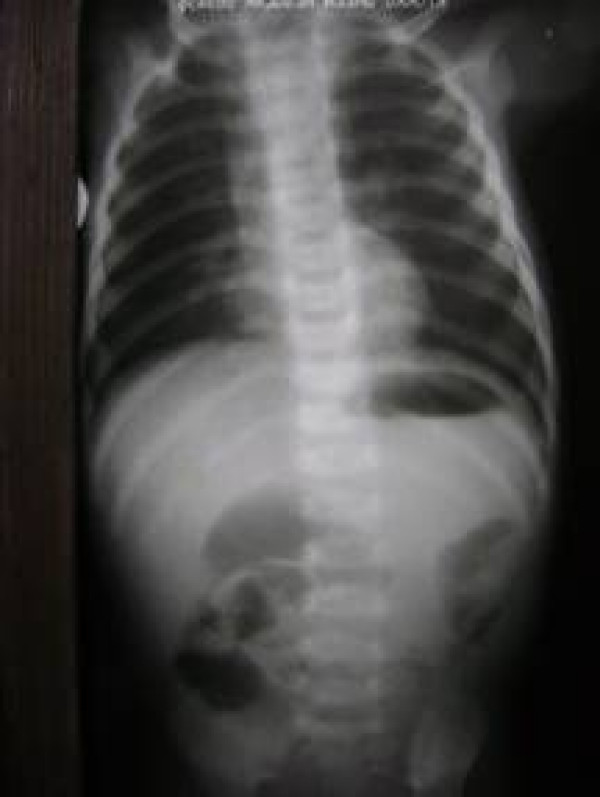
plain x-ray abdomen shows multiple fluid level.

**Figure 2:**
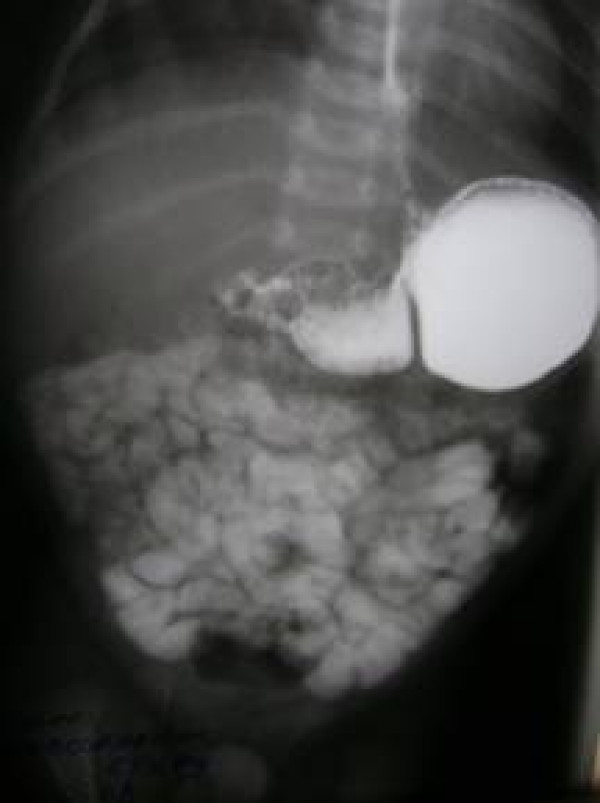
upper GI contrast study shows prepyloric stricture.

Plain x-ray abdomen showed air fluid level [figure 1]. The upper GI contrast study showed a dilated double loop of stomach with significant constriction at prepyloric region with delayed gastric emptying. [Figure 2]

**Figure 3:**
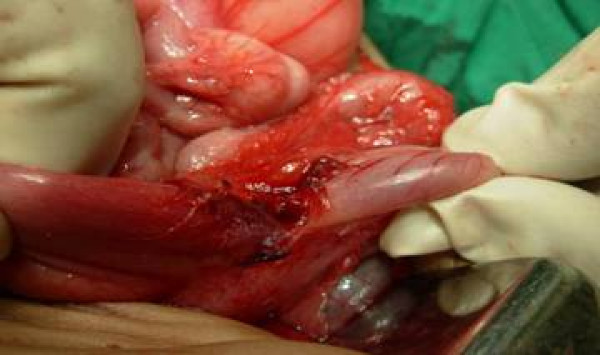
shows prepyloric stricture of stomach.

The diagnosis of prepyloric stricture was made and baby was planned for elective laparotomy. On exploration the stomach was distended with stricture in prepyloric region which was covered by greater omentum [figure 3]. Longitudinal incision was made over the stricture segment along the line of pylorus and transversally stitched. [fig, 4,] The patency was checked by passing normal saline through the segment. The post-operative period was uneventful and a patient is doing well in follow-up.

**Figure 4:**
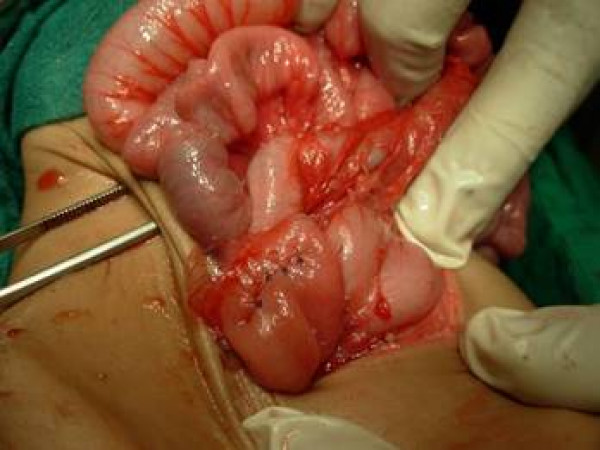
shows stomach after stricturoplasty.

## Discussion

Gastric outlet obstruction (GOO) is a classic indication for surgery in complicated peptic ulcer disease. [1] The gastric outlet obstruction in infancy is a relatively rare condition with a incidence of 1 in 100,000 live births when the IHPS is excluded [2,3]. The incidence of IHPS is 1.5 to3 per 1,000 live births [2]. However the conditions like; gastric-web, pyloric atresia, ectopic pancreatic tissue and duplication of pylorus can also produce the sign symptoms of GOO. The acquired cause of GOO in infants are acid peptic disease, neoplasm and caustic ingestion' [4,5]. With an increase in the incidence of peptic ulceration in pediatric population, complications like gastro-duodenal perforation and pre-pyloric stricture are also encountered [6]. Generally, the course of peptic ulcer in children is longer than that in adults, so the degree of stricture, secondary to long-standing peptic ulceration, in children may be very significant despite the advances in medical management but it usually presents in late childhood. So, medical therapy for children with GOO secondary to peptic ulcer cannot be expected to relieve the obstruction component [7].

Although there are few reports of GOO due to prolapsing gastric polyp in adults, but no report of GOO secondary to antral mass combined with gastric ulcer was found in pediatric age group [8]. Sometimes inflammatory polyp might be resulted from H. pylori infection.[9] Although there are few cases of peptic ulcer perforation had been reported [10,11] but pre-pyloric stricture due to peptic ulcer had not been reported. The GOO due to healed peptic ulcer, perforation and malignancy are seen in adult but rarely seen in pediatric age group. In peptic ulcer diseases, GOO is usually caused by a combination of edema, spasm, fibrotic stenosis and gastric atony.[12] Chan et al. reported their experience with 32 children with duodenal ulcers.[13] GOO caused by peptic ulcer diseases can be resolved by medical treatment,[12]vagotomy, pyloroplasty [12] or endoscopic balloon catheter dilatation.[14,15] However GOO secondary to gastric ulcer in infant had not been reported. As per our knowledge this is the first case report of prepyloric stricture presented as GOO.

## Conclusion

There are several rare causes of GOO in children that may present with a variety of symptoms. Evaluation of such patients may require a wide range of diagnostic studies. Different therapeutic modalities based on the specific cause and degree of obstruction is usually needed to manage this heterogeneous group children

## Competing interests

The authors declare that they have no competing interests.

## Authors' contributions

SPS and PS operated the patient and reviewed the literature. VDU and RJ were the main writers of the manuscript, where as ANG and VK moderated the manuscript. All authors read and approved the final manuscript.

## Consent

Written informed consent was obtained from the parents of the patient for publication of this case report and accompanying images. A copy of the written consent is available for review by the Editor-in-Chief of this journal.
